# Offspring size at weaning affects survival to recruitment and reproductive performance of primiparous gray seals

**DOI:** 10.1002/ece3.1450

**Published:** 2015-03-04

**Authors:** William D Bowen, Cornelia E den Heyer, Jim I McMillan, Sara J Iverson

**Affiliations:** 1 Bedford Institute of OceanographyDartmouth, Nova Scotia, B2Y 4A2, Canada; 2 Department of Biology, Dalhousie UniversityHalifax, Nova Scotia, B3H 4J1, Canada

**Keywords:** *Halicheorus grypus*, life history, marine mammal, pinniped, reproduction

## Abstract

Offspring size affects survival and subsequent reproduction in many organisms. However, studies of offspring size in large mammals are often limited to effects on juveniles because of the difficulty of following individuals to maturity. We used data from a long-term study of individually marked gray seals (*Halichoerus grypus*; Fabricius, 1791) to test the hypothesis that larger offspring have higher survival to recruitment and are larger and more successful primiparous mothers than smaller offspring. Between 1998 and 2002, 1182 newly weaned female pups were branded with unique permanent marks on Sable Island, Canada. Each year through 2012, all branded females returning to the breeding colony were identified in weekly censuses and a subset were captured and measured. Females that survived were significantly longer offspring than those not sighted, indicating size-selective mortality between weaning and recruitment. The probability of female survival to recruitment varied among cohorts and increased nonlinearly with body mass at weaning. Beyond 51.5 kg (mean population weaning mass) weaning mass did not influence the probability of survival. The probability of female survival to recruitment increased monotonically with body length at weaning. Body length at primiparity was positively related to her body length and mass at weaning. Three-day postpartum mass (proxy for birth mass) of firstborn pups was also positively related to body length of females when they were weaned. However, females that were longer or heavier when they were weaned did not wean heavier firstborn offspring.

## Introduction

Studies in a number of taxa show that larger offspring have greater early survival than smaller ones (reviewed in Krist 2011). A major hurdle to understanding the evolution of offspring size is the gap between theory and ecological studies of the effects of offspring size on fitness (Dias and Marshall 2010). Life history theory assumes an offspring size/number trade-off and an offspring size/performance relationship, such that maternal fitness is maximized by the investment strategy that maximizes the number of offspring that survive to reproduce (Smith and Fretwell 1974). Estimating the relationship between offspring size and offspring fitness, however, is challenging for many organisms (Rollinson and Hutchings 2013). Even in mammals and birds where offspring are relatively large and few and individual offspring can be tracked, estimates of the effect of offspring size on subsequent lifetime performance are available for only a small number of taxa (e.g., Clutton-Brock 1991; Festa-Bianchet et al. 2000). Conclusions drawn from few well-studied taxa may not apply generally because trade-offs regarding offspring size and number or performance may depend on species developmental, physiological and behavioral characteristics (Stearns 1992). Thus, field studies of other taxa are needed to provide comparative data to further test the effects of offspring size on both maternal and offspring fitness.

Offspring size effects are known to diminish over time, such that early estimates of effects may result in overestimates of the subsequent optimal size (Heath et al. 1999; Lindholm et al. 2006). One mechanism for this diminished effect is compensatory growth, whereby initial differences among offspring are reduced due to increases in the relative growth rate of smaller offspring (Wilson et al. 2007; Dias and Marshall 2010). Other sources of variation in early development include maternal and paternal effects that can cause cohort differences in various fitness components (Lindstrom 1999). Further, size-selective mortality may depend both on the absolute size and relative size of an individual (Sogard 1997). There may also be ontogenetic changes in the strength and direction of size selection (Sogard 1997). Thus, using estimates of the relationship between offspring size and offspring performance based on early life history stages alone could result in error in both strength and direction of the relationship (Dias and Marshall 2010).

The early effects of offspring size on subsequent fitness (or short-term proxies of fitness) of marine mammals have been investigated in only a few species (reviewed in Bowen 2009). Heavier gray seal (*Halichoerus grypus*) pups and those with higher body condition were shown to have greater survival probability through the first year of life (Hall et al. 2001). Weaning mass and survival through the first year of life were also positively correlated in both southern elephant seals (*Mirounga leonina*) (McMahon et al. 2000) and Hawaiian monk seals (*Monachus schauinslandi*) (Baker 2008). Pup mass at weaning positively affected survival of male, but not female, northern fur seal (*Callorhinus ursinus*) pups through two years of age (Baker and Fowler 1992). Early development traits, such as growth rate, affected short-term postweaning survival in subantarctic fur seals (*Arctocephalus tropicalis*) (Beauplet et al. 2005). Apparent survival from weaning to age 3 year in Weddell seals (*Leptonychotes weddellii*) was positively related to body mass at weaning (Proffitt et al. 2008).

Although the effects of offspring size on early survival have been studied, less is known about how those effects are manifested in adults recruiting to the breeding population (Festa-Bianchet et al. 2000). Attempts to fill this gap are important, as they should provide a better understanding of the fitness consequences of variation in offspring size. However, the relationships between offspring size at weaning and subsequent size and age at primiparity in pinnipeds are unknown.

Gray seals are iteroparous, capital breeders, with indeterminate growth, and precocial young. Twins rarely occur and a litter size of one is usual, as is the case for most pinnipeds and cetaceans. Therefore, offspring size is more variable than offspring number in these species. Primiparity in gray seals typically occurs between the ages of 4 and 7 year and females continue to reproduce into their late 30s (Pomeroy et al. 1999; Bowen et al. 2006). Adult females fast during a brief lactation period averaging 17 day, during which pups more than triple their birth mass consuming energy-rich milk containing 60% lipid during mid-late lactation (Fedak and Anderson 1982; Iverson et al. 1993). Lipids deposited in the form of blubber comprise 40% of the body mass of pups at weaning (Mellish et al. 1999). Weaning is abrupt with the females leaving the colony to feed at sea, while their pups undergo a postweaning fast of several weeks before going to sea (Noren et al. 2008). During this fasting period, pups draw energy mainly from lipids stored during lactation to support metabolic requirements and some physiological growth (Reilly 1991; Noren et al. 2008).

In this study, we tested the bigger-is-better hypothesis that larger female offspring survive better to recruitment, breed at a younger age, are larger at primiparity, and produce larger offspring. We used parturition date and body mass at birth and weaning of firstborn offspring as measures of female performance in free-ranging gray seals.

## Materials and Methods

Our study was conducted between 1998 and 2012 on Sable Island (43°55′ N, 60°00′ W), a partially vegetated sandbar located on the Scotian Shelf approximately 160 km off the east coast of Nova Scotia, Canada. At this colony, gray seal females give birth between the first week of December and late January, but 97% of pups are born by the middle of January (Bowen et al. 2007). The number of pups born on the Island increased exponentially at a rate of 12%/year through the late 1990s (Bowen et al. 2007). Between the late 1990s and 2010, when most of our study females had recruited, the pup production continued to increase but at a reduced rate of 4% (Bowen et al. 2011). Thus, individuals in our study experienced an increasing density of adults in the breeding colony and possibly at sea as well.

Our data were collected from a marked sample of female offspring (MO), a subset of which survived to recruit to the breeding population, which in turn produced their own firstborn (FB) pup. To clarify the text, we refer to these groups of individuals and measurements taken as follows: marked offspring length and mass at weaning (MO_lw,_ MO_mw_); primiparous body length (P_l_) and body mass at 3-day postpartum (P_m3d_), and firstborn pup mass at day 3 postpartum and at weaning (FB_md3;_ FB_mw_). To examine the relationships between offspring size and subsequent survival to recruitment, each year from 1998 to 2002, recently weaned female pups were sedated with diazepam (∽0.4 mg/kg body mass; Sandoz Canada, Boucherville, Quebec, Canada) and permanently marked with a unique three-character brand that permitted identification of individuals over the course of their life (Bowen et al. 2006). Standard dorsal body length (to the nearest cm) was recorded for all pups while they were sedated, allowing accurate length measurements to be taken. Body mass at the time of weaning (to nearest 0.5 kg) was measured only in a subset of these pups that had known weaning dates (see below), as gray seal pups lose mass each day during a postweaning fast (Reilly 1991; Noren et al. 2008).

Apparent survival of offspring from weaning to recruitment was estimated from sightings of the uniquely marked females, ages 4 year or older, during successive breeding seasons on Sable Island. Branded females are rarely seen as 1- to 3-year olds during the breeding seasons precluding a mark–recapture analysis of age-specific juvenile survival. The earliest age of first birth in this population is 4 year (Bowen et al. 2006). The presence of a female in the breeding colony was determined from approximately weekly, whole-island censuses conducted over the course of the breeding season each year (Bowen et al. 2006). Five to seven censuses were conducted from mid-December to the end of January by seven to 10 researchers using all-terrain vehicles. Researchers systematically covered the entire colony in 2–3 days, with the objective of sighting all females. The distance moved by females with pups from 1 day to the next averages about 5 m (Boness and James 1979), such that traveling by females is not a source of sighting error. The first year a female was observed either pregnant or with a pup was operationally defined as the year of recruitment and used to calculate age of primiparity. However, as sighting probability is less than 1.0 (see below) and gray seals do breed elsewhere, it is possible that we could have missed the first birth of some females and therefore overestimated their age at first birth. As about 85% of gray seal females in eastern Canadian waters give birth at our study site, the impact of females breeding elsewhere on mean age of primiparity is expected to be small.

Despite the objective of sighting all marked females on the island during the weekly censuses, some females may not be sighted. There are three ways in which an adult female may not be sighted in a season – she may not be present at the breeding colony – she may be present but not sighted in any of the weekly censuses– she may be present, but seen with an unreadable brand. An open robust mark–recapture analysis (e.g., Schwarz and Stobo 1997) could be used to estimate the probability of sighting females present on the breeding colony as well as the proportion pupping in a year (assessing temporary emigration), but with both high sighting probability and high pupping probability, it is reasonable to expect that those females that survived and established Sable Island as a breeding colony would be seen with 3 to 10 years of resighting effort. A mark–recapture analysis of females from the 1998–2002 cohorts, using the POPAN model, estimated the average sighting probability of 0.66 (den Heyer et al. 2013). Sighting probability accounts for both temporary emigration (females not pupping that year) and probability of observing a female given she returned to the island. Although some females will be missed in any one breeding season, over the multiple years of observation in this study, there is a small chance of missing a female and thus our estimated of apparent survival should be reliable.

Lost or indistinct brands would bias our assessment of apparent survival, but should not change the relationship between survival probability and offspring traits, the objective of our study. In the 1998–2002 branded cohorts, 3.7% or 170 of 4569 sightings (from breeding seasons 2002 to 2012) were not readable either because of a poor quality brand or poor conditions. If roughly 4% of the females that survived from the 1998–2002 cohorts had poor quality marks, the apparent survival rate of roughly 30% would be underestimated by 1.2%. There is no reason to expect that the brand quality would be associated with offspring size, and therefore, loss of brands should not influence our conclusions.

Once sighted, the age/pelage stage of the female's pup (Kovacs and Lavigne 1985; Bowen et al. 2003) was recorded along with the pair's location (using GPS) within the colony, and the pup was given a uniquely numbered hind-flipper tag so that it could be identified after the female left the colony at the end of lactation. Pups were classified as newborn (i.e., <24 h of birth) by the nearby presence of the placenta, the yellowish hue of the pup's white lanugo coat, loose folds of skin along the trunk of the body, and awkward movements (Kovacs and Lavigne^1985^). The age of slightly older pups (1–2 day) was estimated from pelage-stage data collected from known-age pups (Bowen et al. 2003). Both the female and her pup were visited daily (but not disturbed) throughout the remainder of lactation. Weaning occurs abruptly in this species with the female departing at sea while the pup remains alone in the colony. As adult females usually weaned their offspring overnight, new solitary pups were considered weaned at midnight of the previous day they were observed. Pups were weighed (to nearest 0.5 kg) on the day of weaning (*n* = 284) or within 2 days postweaning (*n* = 25).

To investigate the relationships between offspring size and subsequent reproductive performance, parturition date, body length and mass of recruiting females and body mass of their firstborn pups were measured. Parturition date was known for many of the primiparous females or could be reasonably estimated for those females with recently born pups (1–2 days old) based on an assessment of pelage color and morphology (see above). We measured the total body length of all recruiting females. Females were sedated using an IV injection of diazepam (∽0.4 mg/kg body mass) to permit an accurate measurement to be taken. Adult females lose about 4 kg of body mass per day during lactation and their pups gain about 2 kg/day (Mellish et al. 1999). Thus, to examine the relationship between maternal size and birth mass of offspring, only females with known parturition dates could be used. A subset of 59 mothers and their pups, with known parturition dates, was weighed at 2- (*n* = 4), 3- (*n* = 48) and 4-day postpartum (*n* = 7). Waiting several days to weigh the pair reduced the risk of maternal abandonment as a result of disturbance (W. D. Bowen unpubl. obs.).

All procedures used on study animals were in compliance with applicable animal care guidelines of the Canadian Council on Animal Care and were approved by The Department of Fisheries and Oceans Animal Care Committee (Protocol numbers 98-57 through 12-08).

### Statistical analyses

Generalized linear models (GLM) and generalized additive models (GAM) were fit to the data in R 3.0.1 (R Development Core Team 2011). Evidence in favor of competing models was evaluated on the basis of lowest Akaike information criterion (AIC), with finite sample correction (AIC_c_), smallest ΔAIC_c_, highest AIC_c_ weights (*w*), and evidence ratios (Burnham and Anderson 2002). A suite of candidate models was developed from the full model. All models having a ΔAIC_c_ < 2 were considered as having some support, but we preferred models with fewest parameters and the highest *w* and therefore highest evidence ratio. Means are presented with standard errors (SE) throughout and results of hypothesis tests were judged significant at *P *< 0·05.

To test for the effects of offspring body mass and length at weaning on apparent survival to recruitment, we fitted GLMs to the sightings on Sable Island using a binomial error distribution (Table S1). As offspring body length and mass are highly correlated (*r* = 0.71, *P* < 0.001, df = 564), they were not used together in the same model. We expected interannual variability in environmental conditions to influence survival, therefore, the year of branding (cohort) was included in the full model as a factor. Cohort was also included in the model examining predictors of FB_mw_ (Table S14 and S16). Sample size was insufficient to include cohort on other analyses. AIC_c_ was used to identify the minimum number of cohort factors to retain in the model.

We tested for nonlinear effects of offspring size on their subsequent survival to recruitment by including mass and length as a quadratic function. Where the preferred model included a quadratic term for offspring size, we fit a GAM using the R package ‘mgcv’ and a Laplace approximation to REML. The GAM fit of mass at weaning on survival between weaning and recruitment suggested an inflection in the neighborhood of 50 kg. We used piecewise linear models with breakpoints between 45 and 55 kg in steps of 0.25 kg to estimate the inflection point. The piecewise model with the lowest AIC_c_ was chosen.

Estimated age at primiparity varied between 4 and 14 year, but was highly skewed (skew = 1.50, D'Agostino skewness test, *P* < 0.001), with 82% of females recruiting at or before age 7. We tested the odds of an individual first giving birth greater than (1) or less than (0) the mean age (6.4 year) as a function of its body length and mass at weaning using a GLM with a binomial error distribution.

To test for the effects of offspring size on reproductive performance of primiparous females, we fitted GLMs (Table S1) to the 3-day body mass of the female's firstborn pup (FB_m3d_), her pup's body mass at weaning (FB_mw_), and to the day she gave birth standardized to December 1 (nominal start of pupping season).

Both pup sex and maternal age are known to influence reproductive performance in this species (Bowen et al. 2006) and were included as explanatory factors. There are strong effects of maternal body mass on offspring size in gray seals (Pomeroy et al. 1999; Bowen et al. 2006). However, it was not possible to weigh a large enough sample of mothers of the marked offspring at the same stage in lactation to account for the effects of maternal mass on offspring survival in the analysis.

## Results

### Offspring size

Between 1998 and 2002, 1182 female pups were permanently marked and body length was measured near weaning. Marked offspring were either selected at ‘random’ from the population (*n* = 856) or were pups of known-age mothers (*n* = 326). Of those marked, 566 were also weighed at weaning, and 309 of this subset had mothers of known age. Female pups born to known-age mothers were slightly longer at weaning (mean MO_lw_ = 110.6 cm vs. 109.5 cm; *t*-test, *P* < 0.001, df = 1180), but not heavier (mean MO_mw_ = 52.1 kg vs. 52.6 kg; *t*-test, *P* = 0.34, df = 564), than the randomly selected female pups born to unknown females.

Mean MO_lw_ varied (ANOVA, *P* < 0.001, df = 4, 1177) among cohorts, with those born in 2001 and 2002 being longer than those born in 1998 through 2000. However, mean MO_mw_ did not differ among cohorts (ANOVA, *P* = 0.11, df = 4, 561).

### Offspring size and apparent survival to recruitment

**Figure 1:**
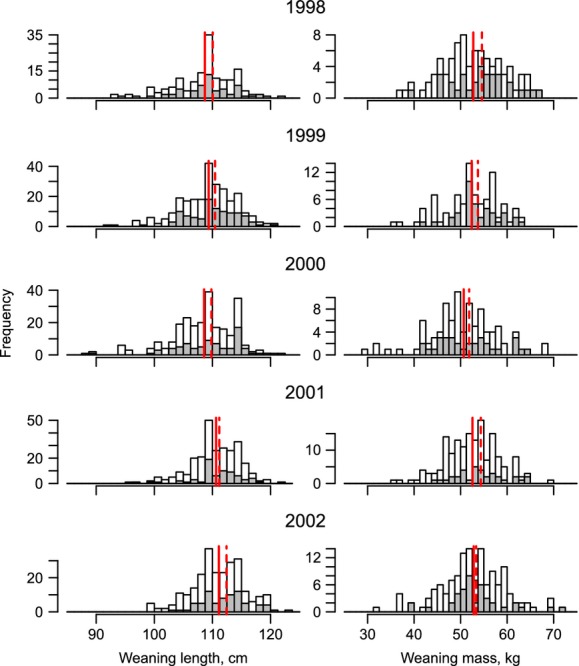
Histograms of body length and mass at weaning for all female gray seals marked between 1998 and 2002 (white) and those subsequently recruited at age 4 year and older (gray). Vertical lines indicate the mean for all females (solid red) and those that were sighted (dashed red).

We addressed two questions with respect to offspring size and apparent survival. The first concerned selection on offspring traits and the second concerned how size influenced apparent survival probability. Regarding the first question, pups that were subsequently sighting in the breeding colony were significantly longer than those that were not sighted in 4 of the 5 years (Table1). Although in all years sighted pups were also heavier than pups that were not sighted, the difference was significant in only 2 of the 5 years (Table1). The frequency distributions of the body length and mass of those pups sighted compared to those marked at weaning are shown in Fig.1.

**Table 1:** Offspring length (MO_lw_) and mass (MO_mw_) of those sighted in the breeding colony compared to those not sighted by cohort. *P*-values based on independent *t*-tests.

Cohort	MO_lw_				MO_mw_			
	Mean sighted	Mean not sighted	df	*P*	Mean sighted	Mean not sighted	df	*P*
1998	110.1	107.9	154	0.012	54.6	51.6	82	0.048
1999	110.4	108.6	256	0.003	53.7	51.4	88	0.068
2000	109.8	108.0	247	0.015	51.8	50.1	94	0.263
2001	111.2	110.4	265	0.199	54.4	52.1	159	0.038
2002	112.4	110.6	250	0.002	53.3	52.6	133	0.556

**Figure 2:**
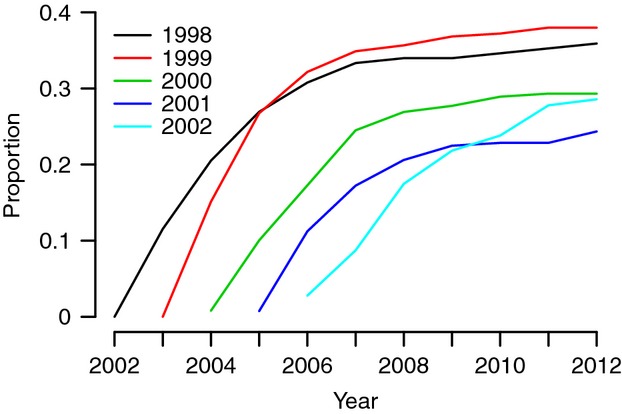
Cumulative proportion of marked female gray seals that recruited to the Sable Island breeding colony by year and cohort.

Of the 1182 marked female offspring, 381 (32%) were resighted for the first time pregnant or with a pup at the Sable Island breeding colony. Only two of the marked females were sighted in other breeding colonies, suggesting that most surviving females recruited to the study area. The proportion of offspring recruiting to the breeding colony as of 2012 varied among cohorts (Table2). Discovery curves of recruiting females appeared asymptotic for all cohorts, excepting perhaps the youngest, and therefore, the proportions of females recruited should accurately reflect year differences in apparent survival (Fig.2). Two additional years of sighting data indicate that our estimates of apparent survival are secure, with no changes in estimates for 1998, 1999, and 2000, and an increase of <1% for the 2001 and 2002 cohorts.

**Table 2:** Cumulative number and percentage of branded females sighted at age 4 year and older during the breeding season each year through 2012.

Cohort	Not sighted	Sighted	% Sighted
1998	98	58	37.2
1999	157	101	39.1
2000	171	78	31.3
2001	198	69	25.8
2002	177	75	29.8
Total	801	381	32.2

**Figure 3:**
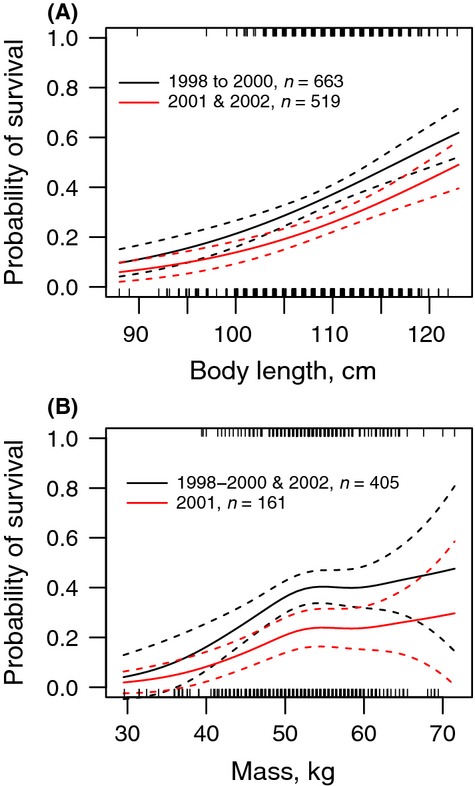
Probability of survival between weaning and recruitment of female gray seals marked as pups on Sable Island predicted from (A) the GAM including body length at weaning and cohort as a factor and (B) the GAM including mass at weaning and cohort as a factor. Rugs show the distribution of the observations.

With respect to the second question, the model that best fit the data on apparent survival to recruitment included MO_lw_ and cohort as explanatory factors (Table3). There was some support for a model that included females’ weaning length as a quadratic function, but the linear model was twice as likely as the quadratic model based on the evidence ratio. Longer offspring had a greater probability of survival than shorter ones for all cohorts (Table4). Model selection based on AIC_c_ (Table S2) indicated the variation associated with the cohort effect could be described by two periods, 1998 to 2000 and 2001 and 2002, with 2001 and 2002 having the lower apparent survival (Fig.3A).

**Table 3:** GLMs of effects of female offspring body length and mass at weaning on probability of subsequent survival to recruitment. Body length (MO_lw_) was measured in all offspring marked and body mass (MO_mw_) was measured from a subset of 566 offspring at weaning.

Model	*N* = 566					*N* = 1182				
	*K*	AIC_c_	ΔAIC_c_	*w*_*i*_	LL	*K*	AIC_c_	ΔAIC_c_	*w*_*i*_	LL
Cohort + MO_lw_	6	686.42	0	0.39	−337.13	6	1453.28	0	0.65	−720.6
Cohort + MO_mw_ + 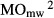	7	687.53	1.11	0.22	−336.66					
Cohort + MO_lw_ + 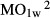	7	687.88	1.46	0.19	−336.84	7	1454.69	1.41	0.32	−720.3
Cohort + MO_mw_	6	689.52	3.1	0.08	−338.69					
Cohort + MO_lw_ + Cohort × MO_lw_	10	693.91	7.49	0.01	−336.75	10	1459.76	6.49	0.03	−719.79
Cohort + MO_mw_ + Cohort × MO_mw_	10	695.07	8.65	0.01	−337.34					
MO_lw_	2	696.48	10.06	0	−346.23	2	1464.79	11.51	0	−730.39
MO_mw_	2	697.16	10.74	0	−346.57					
Cohort	5	698.31	11.89	0	−344.1	5	1482.93	29.66	0	−736.44
Intercept	1	705.27	18.85	0	−351.63	1	1488.05	34.78	0	−743.03

AIC_c_, Akaike Information Criterion for small sample sizes; ΔAIC_c_, relative change in AIC_c_, *w*_*i*_, AIC weights; K, number of parameter.

**Table 4:** Parameter estimates from GLM of the probability of apparent survival of branded female pups as a function of three cohort groups, 1998 to 2000 and 2001 & 2002, and body length at weaning (MO_lw_).

Coefficients	Estimate	SE	*z*-value	Pr(>|z|)
(Intercept)	−9.64	1.573	−6.13	<0.001
2001 & 2002	−0.60	0.165	−3.64	<0.001
MO_lw_	0.08	0.014	5.49	<0.001

Null deviance: 1486.1 on 1181 degrees of freedom.

Residual deviance: 1444.0 on 1178 degrees of freedom.

Apparent survival probability of female offspring also increased with increasing MO_mw_, but there appeared to be an inflection point (Fig.3B). The preferred piecewise linear regression model indicated that the probability of survival increased for heavier offspring until about 51.5 kg, beyond which survival was independent of body mass at weaning (Table5). AIC_c_ (Table S3) indicated that the cohort effect on the probability of survival could be described as two periods (2001 and all other cohorts). The probability of survival was significantly lower for the 2001 cohort than other cohorts. Overall, our results showed that female offspring shorter than 90 cm or those weighing less than 30 kg at weaning had little chance (<10%) of recruiting (Fig.3A and B).

**Table 5:** Parameter estimates from GLM of the probability of apparent survival of branded female pups as a function of two cohort groups, 1998 to 2000 and 2001 & 2002, and body mass at weaning (MO_mw_) with a breakpoint of 51.5 kg, above which survival was independent of mass at weaning.

Coefficients	Estimate	SE	*z*-value	Pr(>|z|)
(Intercept)	−0.89	0.141	−6.32	<0.001
I((MO_mw_ -51.5)*(MO_mw_ <51.5))	0.12	0.032	3.79	<0.001
2001 & 2002	0.65	0.187	3.49	<0.001

Null deviance: 703.27 on 565 degrees of freedom.

Residual deviance: 675.09 on 563 degrees of freedom.

### Offspring size vs. age and size at primiparity

Although 381 females were estimated to have been primiparous on first sighting, limited time and personnel during short winter days meant that not all of these females and their pups could be captured and measured. Therefore, sample sizes available to test hypotheses concerning the influence of MO traits on the traits of primiparous females and their firstborn offspring are less than the total number of primiparous females observed.

**Figure 4:**
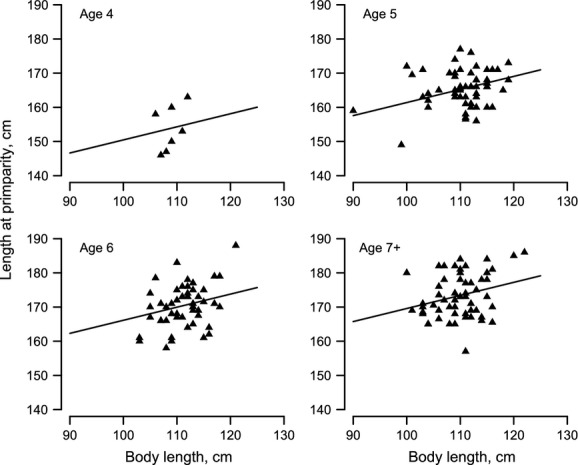
Maternal length as a function of body length at weaning (MO_lw_) and age at primiparity (P_l_). See Table5 for regression coefficients.

There was no significant difference in mean age of primiparity among cohorts (ANOVA, *P* = 0.53, df = 4, 325). Neither MO_mw_ (*P* = 0.68, df = 152) nor MO_lw_ (*P* = 0.74, df = 328) differed significantly among those females that had their first pup at ages 4 and 5 compared to 6 year and older. MO_lw_ explained 6% of the variance in body length at primiparity (P_l_), with relatively longer offspring also being longer at age of primiparity (Fig.4, Table6). As expected, older primiparous females were also longer (different intercepts), but the relationship with MO_lw_ was the same across ages (constant slopes, Table S4). MO_mw_ explained only 3% of the variance in P_l_ (Fig. S5, Table S6), with the same four age groups contributing to the explained variance (Table S7). However, neither MO_lw_ (*n* = 56, Table S8) nor MO_mw_ (*n* = 29, Table S9) explained the variation in the P_m3d_. Given the small samples sizes used for these analyses, more data will be needed to confirm these results.

**Table 6:** Parameter estimates from GLM of length at age of primiparity (P_l_) as a function of body length at weaning (MO_lw_) and age at primiparity (ages 4, 5, and 6 and 7+ year).

Coefficients	Estimate	SE	*z*-value	Pr(>|z|)
(Intercept)	112.0	10.8	10.4	<0.001
MO_lw_	0.4	0.1	3.9	<0.001
Age 5	10.9	2.3	4.7	<0.001
Age 6	15.7	2.3	6.7	<0.001
Age 7+	19.1	2.3	8.3	<0.001

Null deviance 9697.5 on 174 degrees of freedom.

Residual deviance: 5626.8 on 170 degrees of freedom.

### Offspring size vs. body mass of firstborn pups

By influencing maternal size, a female's size when she was weaned might also influence the size of her firstborn pup. To test this, we measured FB_m3d_ (proxy for birth mass) and FB_mw_. The preferred model of FB_m3d_ included MO_lw_ (Table S11), although this trait explained only 7% of the variation. When MO_mw_ was used rather than MO_lw_, the preferred model of FB_m3d_ included only pup sex (Table S12). Male pups were larger than female pups, but only 10% of the variance in FB_m3d_ was explained by sex.

The FB_mw_ was measured of 249 females whose length was measured when they were offspring and 113 females where both length and body mass were taken. The best model for the 249 females explained 25% of variation in the FB_mw_ and included pup sex, maternal age, and MO_lw_ (Table S13, S14, and S15), but the confidence limit on the estimate of MO_lw_ included zero, indicating the this factor was uninformative. The model for the 113 females that best explained variation in the weaning mass of the firstborn pup included only pup sex and maternal age (Table S16). This model explained 33% of the observed variation. Thus, there was little evidence that the measured offspring traits of the mother influenced the weaning mass of her firstborn pup.

### Parturition date of recruiting females

Primiparous females gave birth from December 12 to January 25, with an overall mean and median birth date of January 1. Earlier birth is associated with greater success in gray seals because females that give birth early are harassed less by adult males (see Boness et al. 1995). The best model explained 13% of variation in the parturition date of primiparous females and included maternal age and MO_lw_ (Table S17, S18). However, the confidence limits for the estimates of the effect of MO_lw_ on parturition date included zero suggesting this factor was uninformative. The effect of maternal age on parturition date was described by three groups (ages 4–6, 7 & 8, and 9+; Table S19). The model with only maternal age also had substantial support (ΔAIC_c_ = 0.57). Restricting the data to mothers for which MO_mw_ was measured, the preferred model included only maternal age (Table S20), with older primiparous females pupping earlier in the breeding season (21% of variance explained). Taken together, these analyses suggest that older mothers gave birth earlier.

## Discussion

Relatively few studies have attempted to link offspring size with subsequent reproductive output for any free-ranging organism (Dias and Marshall 2010; Krist 2011). In gray seal females, larger offspring had a greater probability of apparent survival to recruitment, but offspring body mass and body length had different effects on survival probability. Recruiting females were longer when they were weaned than those that did not recruit, suggesting size-selective mortality. Offspring size (both mass and length at weaning) was weakly correlated with a female's subsequent body length at recruitment; however, it did not appear to influence her age or body mass at recruitment. A female's size when she was weaned also positively affected the birth mass of her firstborn pup. Older primiparous gray seals gave birth earlier in the breeding season, but parturition date were not influenced their size at weaning.

Our estimates of apparent survival assume that being branded does not confer an increased risk of mortality and that marked females that returned to the Island are observed. Although we do not have estimates in gray seals, studies using similar methods to ours indicate that branding does not significantly increase mortality in other pinniped species (Hastings, Gelatt & King 2009; McMahon et al. 2006; Wilkinson et al. 2011). Gray seals exhibit a high degree of philopatry, and there are only a few other breeding colonies of NW Atlantic gray seals. Of the females branded between 1998 and 2002, only 2 have been sighted at breeding colonies other than Sable Island. Therefore, emigration of branded females should not have significantly biased our results. Our estimates of apparent survival and age at primiparity are influenced by sighting probability. A mark–recapture analysis of females from the 1998–2002 cohorts estimated the average sighting probability of 0.66 (den Heyer et al. 2013). Over multiple years (*n*) of observation, there is a small chance (0.34^*n*^) that we missed a female that established Sable Island as her breeding site. For example, the case of females that were branded in 2002 (the youngest cohort) and recruited to island at age 6 (2008), there would have been 5 years of possible resightings, thus less than 1% chance we would have missed her. Therefore, our data should provide reliable estimates of apparent survival.

Offspring size effects may be highly context dependent. For example, survival and reproductive success are expected to vary with population density and the availability of per capita food resources (e.g., Wilson et al. 2005a,b). Female gray seals born during a period of exponential population growth in the 1980s and 1990s (Trzcinski et al. 2006) had apparent juvenile survival probabilities to age 4 year of 0.7–0.8 (Schwarz and Stobo 2000). By contrast, during the 2000s, our study females experienced reduced population growth rate as the population approached carrying capacity (Bowen et al. 2007, 2011), with correspondingly lower apparent juvenile survival probabilities ranging from 0.26 to 0.39 through the 2012 breeding season (this study and den Heyer et al. 2013). As selection on traits is predicted to increase in the face of increasing resource limitation (e.g., de Little et al. 2007; Baker 2008), our estimates of the effects of offspring size may be greater than during other periods.

Studies in a number of taxa show that larger offspring have greater early survival than smaller ones (e.g., Van Ballenberghe and Mech 1975; Guinness et al. 1978; reviewed in Krist 2011). Fewer studies have tested the effects of offspring size on survival to recruitment and reproductive performance (Coltman et al. 1999; Beauplet et al. 2006; Uller and Olsson 2010). Our results showed that gray seal females that survived to recruitment and were sighted on Sable Island during the breeding season were, on average, 1.5 cm longer as weaned offspring than females that were not sighted lending further support to the bigger-is-better hypothesis.

Generally, an asymptotic relationship between offspring size and survival is predicted because parents should receive decreasing returns on offspring fitness with increasing offspring size (Smith and Fretwell 1974; Dias and Marshall 2010; Jorgensen et al. 2011). In species producing few large offspring, such as marine mammals and seabirds, fewer parents are predicted to produce offspring near the physiological limit of viability and hence, the offspring fitness curve may approach the minimum viable offspring size relatively slowly (Rollinson and Hutchings 2013). Our results and those of Hall et al. (2001) provide some support for this expectation in gray seals. Hall et al. (2001) found an asymptotic relationship between first-year survival and both body mass and body condition at weaning. Similarly, Baker (2008) found an asymptotic relationship between first-year survival and offspring girth in Hawaiian monk seals. Over a longer period (i.e., to recruitment), we found that body mass at weaning was positively related to survival for lighter pups, but for pups heavier than 51.5 kg (mean weaning mass in the population), there was no further effect of body mass (Fig.3). Furthermore, the offspring fitness curve approached the minimum viable offspring size relatively slowly in both of these gray seal studies, as did pup growth rate in subantarctic fur seals (Beauplet et al. 2005).

The model of juvenile survival as a function of body length at weaning was preferred over the model using body mass at weaning. We speculate that this might reflect the survival advantage of larger skeletal size which may increase foraging ability. Young of the year gray seals do make longer foraging trips and forage farther from haul-out sites than older animals (Breed et al. 2011), indicating that greater effort associated with foraging could reflect ineffective foraging behaviors (Marchetti and Price 1989). Longer pups may also be better equipped to avoid predators (Hindell et al. 1999). However, both of these hypotheses remain untested in gray seals.

Although offspring body mass and length both influenced survival to recruitment in gray seals, the shape of the relationships differed. Survival increased with body mass to an asymptote near observed average weaning mass in this population (Bowen et al. 2006), suggesting stabilizing selection on body mass. This contrast with studies of Soay Sheep (*Ovis aries*) which showed strong direction selection on offspring mass (Wilson et al. 2005a,b). By contrast, survival increased monotonically with body length, suggesting directional selection and supporting the bigger-is-better hypothesis. Why should selection on these traits differ? Newly weaned gray seal offspring comprises up to 46% fat in the form of blubber (Mellish et al. 1999). This energy-rich depot is catabolized by offspring during transition to independent foraging. Thus offspring with larger energy reserves (i.e., larger body mass) may fare better as they have greater short-term insurance against starvation that influences survival in the first few months postweaning (also see Baker 2008). That much beyond the population average weaning mass there appears to be little survival benefit suggests there may be costs associated with being too heavy. One possibility is that heavier/fatter pups are more buoyant (Beck et al. 2000) and therefore may be more vulnerable to predation or less efficient foragers. Both of these speculations remain to be tested.

Body length, however, is a better measure of overall skeletal size which can be expected to confer a more enduring advantage in foraging and perhaps predator avoidance by outgrowing the predator over the first few years of life. Mortality generally decreases with increasing body size (Peterson and Wroblewski 1984), and it is widely assumed that rapid growth enhances survival by allowing individuals to escape predators (Sogard 1997). Shark predation is a source of mortality in juvenile gray seals (Brodie and Beck 1983), but the importance of this predation over the first few years of live is not known. Nevertheless, our study does suggest that conclusions about selection on body size may differ when different metrics of body size are considered. These differences may reflect ontogenetic changes in the factors influencing the correlation between body size and survival.

We found significant cohort or year–class effects of offspring traits on female survival to recruitment, with most females recruiting between 5 and 8 years of age. Cohort effects on life-history traits are well documented in seabirds (Boersma and Parrish 1998), ungulates (Gaillard et al. 1997), and pinnipeds (Beauplet et al. 2005; de Little et al. 2007). Interannual variation in survival is often attributed to environmental variation or density effects on food availability (e.g., Post and Stenseth 1999). However, the cause of this variation in our study is unknown and will be difficult to examine given the long period over which survival is determined. In several other phocid species, the greatest mortality occurs in the first 2 years of life (Hastings et al. 1999; Baker and Thompson 2007), but age-specific survival rates are unknown in gray seals so it is not clear at what age environmental drivers of variation may operate. Nevertheless, the effects of environmental variation on the relationship between offspring size and survival are evident from our data even though the underlying cause and timing are unknown.

It is expected that the effects of maternal investment in offspring size is most significant at early life-history stages (Dias and Marshall 2010), with compensatory growth or other factors reducing impact later in life (e.g., domestic sheep, Wilson & Réale 2005, red squirrels (*Sciurus vulgaris*, Wauters et al. 1993). Our study demonstrated that offspring characteristics such as length and mass strongly affect the probability of survival to adulthood, but as expected, effects at primiparity were greatly reduced. Offspring size had no detectable influence on age at recruitment. Nevertheless, large female offspring had better survival and were both longer and heavier at primiparity than smaller ones, as also reported in lizards (Uller and Olsson 2010). Females that were larger at weaning also gave birth to heavier pups, but the effects were small in all cases. Further data are needed to evaluate the longer term influence of offspring size on reproductive success, but studies on terrestrial mammals have shown that larger surviving offspring have higher lifetime reproductive success (Wilson et al. 2005a,b). Beauplet and Guinet (2007) found that estimates of lifetime reproductive success in subantarctic fur seals were positively correlated with measures of female quality. It remains to be determined if offspring size also correlates with female fitness in gray seals.

## Supporting Information

**Table S1.**Generalized linear models (GLM) to test offspring-size effects on subsequent survival to recruitment as a function the following covariates: MO_lw_ = offspring body length at weaning, MO_mw_ = offspring mass at weaning, cohort (factor), cohort (factor), p_rec_ = probability of recruiting after mean age primiparity, P_l_ = body length at age of primiparity, P_m3d_ = body mass 3-days postpartum at primiparity, FB_m3d_ = pup mass 3-days postpartum, FB_mw_ = firstborn mass at weaning, FB_bd_ = first born birth date, momage = maternal age (factor), pupsex = pup sex (factor). **Table S2.** AIC_c_ model selection for cohort groups to test the preferred model with offspring body length at weaning (surv ∽ MO_lw_ + cohort). **Table S3.** AIC_c_ model selection for cohort groups to determine the preferred model with offspring mass at weaning (surv ∽ MO_mw_ +  + cohort). **Table S4**. AIC_c_ model selection for the model of maternal length at age of primiparity as a function of offspring length at weaning (MO_lw_) and age of primiparity (P_l_ ∽ momage + MO_lw_). **Fig. S5.** Maternal length at age of primiparity as a function of mass at weaning and age at primiparity. **Table S6.** Analysis of deviance table for GLM of length at age of primiparity (P_l_) as a function of body length at weaning (MO_lw_) and age at primiparity (ages 4, 5 and 6 and 7+ year). **Table S7**. AIC_c_ model selection for age groups for the model of maternal length at age of primiparity as a function of offspring mass at weaning and age of primiparity (P_l_ ∽ momage + MO_mw_). **Table S8.** AIC_c_ model selection for primiparous maternal mass (P_m3d_) as a function of maternal age at primiparity and offspring length at weaning (MO_lw_, *n* = 56). **Table S9.** AIC_c_ model selection for primiparous maternal mass (P_m3d_) as a function of maternal age at primiparity and offspring mass at weaning (MO_mw_, n = 29). **Table S10.** Parameter estimates from GLM of first-born pup mass at 3 days postpartum (FB_m3d_) as a function of recruited females’ length at weaning (P_l_). **Table S11.** AIC_c_ model selection for first-born pup mass at 3-days postpartum (FB_m3d_) as a function of offspring body length at weaning (MO_lw_, *n* = 58). **Table S12.** AIC_c_ model selection for first-born pup mass 3 days postpartum (FB_m3d_) as a function of offspring length (MO_lw_) or mass at weaning (MO_mw_, *n* = 29). **Table S13.** Parameter estimates from GLM of primiparous pup mass at weaning (FB_mw_) as a function of maternal age (ages 4 & 5, 6, 7 and 8+ year), pup sex and recruited females’ length at weaning (MO_lw_). **Table S14.** AIC_c_ model selection for first-born pup mass at weaning (FB_mw_) and pup sex, cohort, maternal age at primiparity, and offspring body length at weaning (MO_lw_ *n* = 249). **Table S15.** AIC_c_ model selection for age groups for first-born pup weaning mass (FB_mw_) as a function of maternal age at primiparity and offspring body length at weaning (MO_lw_) (FB_mw_ ∽ momage + MO_lw_). **Table S16.** AIC_c_ model selection for first-born pup mass at weaning (FB_mw_) as a function of pup sex, mother age, and offspring length (MO_lw_) or mass at weaning (MO_mw_, *n* = 113). **Table S17.** Parameter estimates from GLM of birth date (days since Dec 1) as a function of maternal age (4–6, 7–8, 9+ year) and recruited female's length at weaning (MO_lw_). **Table S18.** AIC_c_ model selection for first-born birth date as a function of mother age, year, and offspring body length at weaning (MO_lw_, *n* = 163). **Table S19.** AIC_c_ model selection for age groups for the model of first-born birth date (since Dec 1) as a function of maternal age at primiparity and offspring body length at weaning (MO_lw_; FP_bd_ ∽ momage + MO_lw_). **Table S20.** AIC_c_ model selection for first-born birth date and mother age, year, and offspring length (MO_lw_) or mass at weaning (MO_mw_, *n* = 78).
