# Supplementary material for: Offspring size at weaning affects survival to recruitment and reproductive performance of primiparous gray seals
Source: Ecol Evol. 2015 Mar 4;5(7):1412–24. doi: 10.1002/ece3.1450 (PMC4395171; doi:10.1002/ece3.1450)
Supplement: Supplementary file 1 — Table S1.Generalized linear models (GLM) to test offspring-size effects on subsequent survival to recruitment as a function the following covariates: MOlw = offspring body length at weaning, MOmw = offspring mass at weaning, cohort (factor), cohort (factor), prec = probability of recruiting after mean age primiparity, Pl = body length at age of primiparity, Pm3d = body mass 3-days postpartum at primiparity, FBm3d = pup mass 3-days postpartum, FBmw = firstborn mass at weaning, FBbd = first born birth date, momage = maternal age (factor), pupsex = pup sex (factor). Table S2. AICc model selection for cohort groups to test the preferred model with offspring body length at weaning (surv ∽ MOlw + cohort). Table S3. AICc model selection for cohort groups to determine the preferred model with offspring mass at weaning (surv ∽ MOmw + + cohort). Table S4. AICc model selection for the model of maternal length at age of primiparity as a function of offspring length at weaning (MOlw) and age of primiparity (Pl ∽ momage + MOlw). Fig. S5. Maternal length at age of primiparity as a function of mass at weaning and age at primiparity. Table S6. Analysis of deviance table for GLM of length at age of primiparity (Pl) as a function of body length at weaning (MOlw) and age at primiparity (ages 4, 5 and 6 and 7+ year). Table S7. AICc model selection for age groups for the model of maternal length at age of primiparity as a function of offspring mass at weaning and age of primiparity (Pl ∽ momage + MOmw). Table S8. AICc model selection for primiparous maternal mass (Pm3d) as a function of maternal age at primiparity and offspring length at weaning (MOlw, n = 56). Table S9. AICc model selection for primiparous maternal mass (Pm3d) as a function of maternal age at primiparity and offspring mass at weaning (MOmw, n = 29). Table S10. Parameter estimates from GLM of first-born pup mass at 3 days postpartum (FBm3d) as a function of recruited females’ length at weaning (Pl). Table S11. AICc model [file ece30005-1412-sd1.docx]

Table S1. Generalized linear models (GLM) to test offspring-size effects on subsequent survival to recruitment as a function the following covariates: MO_lw_ = offspring body length at weaning, MO_mw_ = offspring mass at weaning, cohort (factor), year of primiparity (factor), p_rec_ = probability of recruiting after mean age primiparity, P_l_ = body length at age of primiparity, P_m3d_ = body mass of primiparous females at 3-days postpartum ; FB_m3d_ = first born pup mass 3-days postpartum, FB_mw_ = first born pup mass at weaning, FB_bd_ = first born birth date, momage=maternal age (factor), pupsex=pup sex (factor).

| Response variable | Error Distribution | Full model |
| --- | --- | --- |
| Survival | Binomial | cohort*MO_lw_, cohort*MO_mw_ |
| p_rec_ | Binomial | MO_lw_, MO_mw_ |
| P_l_ | Gaussian | MO_lw_*momage, MO_mw_*momage |
| P_m3d_ | Gaussian | MO_lw_*momage, MO_mw_*momage |
| FB_m3d_ | Gaussian | MO_lw_+momage+pupsex, MO_m_ +momage+pupsex |
| FB_mw_ | Gaussian | MO_lw_+momage+pupsex, MO_mw_+momage+pupsex |
| FB_bd_ | Gaussian | MO_lw_+cohort+momage, MO_mw_+cohort+momage |
|  |  |  |

Table S2. AIC_c_ model selection for cohort groups to test the preferred model with offspring body length at weaning (surv ~ MO_lw_ + cohort). Preferred model is in bold.

| Cohort Factors | *K* | AIC_c_ | ∆AIC_c_ | *w_i_* |
| --- | --- | --- | --- | --- |
| **1998-2000,2001&2002** | **3** | **1450.71** | **0.00** | **0.49** |
| 1998-2000,2001,2002 | 4 | 1452.08 | 1.37 | 0.25 |
| 1998,1999,2000,2001,2002 | 6 | 1453.28 | 2.57 | 0.14 |
| 1998,1999&2000,2001,2002 | 5 | 1453.81 | 3.10 | 0.10 |
| 1998-2000&2002,2001 | 3 | 1457.48 | 6.77 | 0.02 |
| 1998-2001,2002 | 3 | 1463.94 | 13.23 | 0.00 |

Table S3. AIC_c_ model selection of cohort groups to include in the model of survival with offspring mass at weaning (surv ~ MO_mw_ +MO_mw_ ^2^ + cohort). Preferred model is in bold.

| Cohort Factors | *K* | AIC_c_ | ∆AIC_c_ | *w_i_* |
| --- | --- | --- | --- | --- |
| **1998-2000,2001,2002** | **5** | **684.19** | **0.00** | **0.36** |
| 1998-2000&2002,2001 | 4 | 684.86 | 0.67 | 0.25 |
| 1998&1999,2000,2001,2002 | 6 | 685.72 | 1.54 | 0.16 |
| 1998-2000,2001&2002 | 4 | 685.82 | 1.63 | 0.16 |
| 1998,1999,2000,2001,2002 | 7 | 687.53 | 3.34 | 0.07 |
| 1998-2001,2002 | 4 | 697.75 | 13.57 | 0.00 |

Table S4. AIC_c_ model selection for the model of maternal length at age of primiparity as a function of offspring length at weaning (MO_lw_) and age of primiparity (P_l_ ~ momage + MO_lw_). Preferred model is in bold.

| Age Factors | *K* | AIC_c_ | ∆AIC_c_ | *w_i_* |
| --- | --- | --- | --- | --- |
| **4,5,6,7+** | **6** | **1116.47** | **0.00** | **0.60** |
| 4,5,6,7,8+ | 7 | 1118.45 | 1.98 | 0.22 |
| 4,5,6,7,8,9+ | 8 | 1119.82 | 3.36 | 0.11 |
| all ages | 9 | 1121.64 | 5.17 | 0.05 |
| 4,5,6+ | 5 | 1124.00 | 7.53 | 0.01 |
| 4,5&6,7+ | 5 | 1132.33 | 15.86 | 0.00 |
| 4&5,6,7,8,9,10 | 8 | 1141.35 | 24.88 | 0.00 |

Fig. S5. Maternal length at age of primiparity as a function of her mass at weaning and age at primiparity.

Table S6. Analysis of deviance table for GLM of length at age of primiparity (P_l_) as a function of body lenght at weaning (MO_lw_) and age at primiparity (ages 4, 5 and 6 and 7+ yr).

|  | df | Deviance | Residual DF | Residual Deviance |
| --- | --- | --- | --- | --- |
| NULL |  |  | 79 | 5060.9 |
| MO_wl_ | 1 | 143.68 | 78 | 4917.2 |
| momage | 3 | 2605.44 | 75 | 2311.7 |

Table S7. AIC_c_ model selection for age groups for the model of maternal length at age of primiparity as a function of offspring mass at weaning and age of primiparity (P_l_ ~ momage + MO_mw_). Preferred model is in bold.

| Age Factors | *K* | AIC_c_ | ∆AIC_c_ | *w_i_* |
| --- | --- | --- | --- | --- |
| **4,5,6,7,8+** | **7** | **507.73** | **0.00** | **0.53** |
| 4,5,6,7+ | 6 | 509.28 | 1.55 | 0.24 |
| 4,5,6,7,8,9+ | 8 | 510.17 | 2.45 | 0.16 |
| all | 9 | 512.13 | 4.41 | 0.06 |
| 4,5&6,7+ | 5 | 516.62 | 8.89 | 0.01 |
| 4,5,6+ | 5 | 521.13 | 13.40 | 0.00 |
| 4&5,6,7,8,9,10 | 8 | 524.88 | 17.15 | 0.00 |

Table S8. Chi-squared test of primiparous maternal mass (P_m3d_) as a function of maternal age at primiparity and offspring length at weaning (MO_lw_, n=56).

| Model | Residual  df | Residual deviance | df | Deviance | P(>\|Chi\|) |
| --- | --- | --- | --- | --- | --- |
| P_m3d_ ~ MO_lw_ * momage | 47 | 10308 |  |  |  |
| P_m3d_ ~ momage | 51 | 11103 | -4 | -794.3 | 0.460 |

Table S9. Chi-squared test of primiparous maternal mass (P_m3d_) as a function of maternal age at primiparity and offspring mass at weaning (MO_mw_, n=29).

| Model | Residual  df | Residual deviance | df | Deviance | P(>\|Chi\|) |
| --- | --- | --- | --- | --- | --- |
| P_m3d_ ~ MO_mw_ *momage | 21 | 4104.4 |  |  |  |
| P_m3d_ ~ momage | 25 | 4624.1 | -4 | -519.64 | 0.617 |

Table S10. Parameter estimates from GLM of first-born pup mass at 3 days postpartum (FB_m3d_) as a function of recruited females’ length at weaning (P_l_).

| Coefficients | Estimate | SE | z-value | Pr(>\|z\|) |
| --- | --- | --- | --- | --- |
| (Intercept) | -4.33 | 10.46 | -0.41 | 0.681 |
| P_l_ | 0.20 | 0.09 | 2.07 | <0.05 |

Null deviance: 556.60 on 57 degrees of freedom

Residual deviance: 516.93 on 56 degrees of freedom

Table S11. AIC_c_ model selection for first-born pup mass at 3-days postpartum (FB_m3d_) as a function of offspring body length at weaning (MO_lw_, n=58). Preferred model is in bold.

| Model | K | AICc | ∆AICc | AICcWi |
| --- | --- | --- | --- | --- |
| **MO_lw_** | **3** | **297.91** | **0.00** | **0.35** |
| pupsex+ MO_lw_ | 4 | 299.19 | 1.27 | 0.18 |
| intercept | 2 | 299.98 | 2.06 | 0.12 |
| momage+ MO_lw_ | 7 | 300.21 | 2.30 | 0.11 |
| pupsex | 3 | 300.54 | 2.62 | 0.09 |
| momage+pupsex+ MO_lw_ | 8 | 300.96 | 3.05 | 0.08 |
| momage+pupsex | 7 | 302.55 | 4.63 | 0.03 |
| momage | 6 | 302.76 | 4.85 | 0.03 |

Table S12. AIC_c_ model selection for first-born pup mass 3 days postpartum (FB_m3d_) as a function of offspring length (MO_lw_) or mass at weaning (MO_mw­_, n=29). Preferred model is in bold.

| Model | K | AICc | ∆AICc | AICcWi |
| --- | --- | --- | --- | --- |
| **pupsex** | **3** | **157.47** | **0.00** | **0.33** |
| intercept | 2 | 157.96 | 0.49 | 0.26 |
| pupsex+ MO_lw_ | 4 | 159.89 | 2.42 | 0.10 |
| MO_lw_ | 3 | 159.89 | 2.43 | 0.10 |
| pupsex+ MO_mw­_ | 4 | 160.08 | 2.61 | 0.09 |
| MO_mw­_ | 3 | 160.40 | 2.93 | 0.08 |
| momage | 5 | 163.79 | 6.32 | 0.01 |
| momage+pupsex | 6 | 164.11 | 6.65 | 0.01 |
| momage+ MO_lw_ | 6 | 165.78 | 8.31 | 0.01 |
| momage+ MO_mw­_ | 6 | 166.11 | 8.64 | 0.00 |
| momage+pupsex+ MO_mw­_ | 7 | 166.44 | 8.97 | 0.00 |
| momage+pupsex+ MO_lw_ | 7 | 166.73 | 9.26 | 0.00 |

Table S13. Parameter estimates from GLM of primiparous pup mass at weaning (FB_mw_) as a function of maternal age (ages 4&5, 6, 7 and 8+ yr), pup sex and recruited females’ length at weaning (MO_lw_).

| Coefficients | Estimate | SE | z-value | Pr(>\|z\|) |
| --- | --- | --- | --- | --- |
| (Intercept) | 12.6 | 13.39 | 0.93 | 0.350 |
| age 6 | 4.1 | 1.30 | 3.13 | <0.01 |
| age 7 | 8.2 | 1.46 | 5.58 | <0.001 |
| age 8+ | 11.8 | 1.52 | 7.75 | <0.001 |
| female | -3.7 | 1.04 | -3.56 | <0.001 |
| MO_lw_ | 0.2 | 0.12 | 1.82 | 0.070 |

Null deviance: 21458 on 248 degrees of freedom

Residual deviance: 16130 on 243 degrees of freedom

Table S14. AIC_c_ model selection for first-born pup mass at weaning (FB_mw_) and pup sex, maternal age at primiparity, year of primiparity, and offspring body length at weaning (MO_lw_ n= 249). Preferred model is in bold.

| Model | *K* | AIC_c_ | ∆AIC_c_ | *w_i_* |
| --- | --- | --- | --- | --- |
| **age+pupsex+ MO_lw_** | **13** | **1764.13** | **0.00** | **0.68** |
| age+pupsex | 12 | 1765.72 | 1.59 | 0.31 |
| age+ MO_lw_ | 12 | 1774.78 | 10.65 | 0.00 |
| age | 11 | 1776.39 | 12.26 | 0.00 |
| age+pupsex+ MO_lw_ +year | 22 | 1777.94 | 13.80 | 0.00 |
| age+pupsex+year | 21 | 1779.31 | 15.18 | 0.00 |
| age+ MO_lw_ +year | 21 | 1787.43 | 23.30 | 0.00 |
| age+year | 20 | 1789.07 | 24.93 | 0.00 |
| pupsex | 3 | 1816.32 | 52.18 | 0.00 |
| MO_lw_ +pupsex | 4 | 1816.90 | 52.77 | 0.00 |
| intercept | 2 | 1820.32 | 56.19 | 0.00 |
| MO_lw_ | 3 | 1820.86 | 56.72 | 0.00 |
| year+pupsex | 12 | 1827.44 | 63.30 | 0.00 |
| pupsex+ MO_lw_ +year | 13 | 1828.52 | 64.39 | 0.00 |
| year | 11 | 1831.03 | 66.90 | 0.00 |
| MO_lw_ +year | 12 | 1831.99 | 67.85 | 0.00 |

Table S15. AIC_c_ model selection for age groups to include in the analysis of first-born pup weaning mass (FB_mw_) as a function of maternal age at primiparity and offspring body length at weaning (MO_lw_) (FB_mw_ ~ momage + MO_lw_). Preferred model is in bold.

| Age Factors | *K* | AIC_c_ | ∆AIC_c_ | *w_i_* |
| --- | --- | --- | --- | --- |
| **4to9+** | **9** | **1758.75** | **0.00** | **0.28** |
| 4&5to8+ | 7 | 1759.67 | 0.92 | 0.17 |
| 4to8+ | 8 | 1759.69 | 0.94 | 0.17 |
| 4to10+ | 10 | 1759.92 | 1.18 | 0.15 |
| 4to11+ | 11 | 1760.85 | 2.11 | 0.10 |
| 4to7+ | 7 | 1762.18 | 3.43 | 0.05 |
| 4to12+ | 12 | 1762.67 | 3.92 | 0.04 |
| all | 13 | 1764.13 | 5.39 | 0.02 |
| 4&5,6&7,8+ | 6 | 1764.59 | 5.84 | 0.01 |
| 4&5&6,7,8+ | 6 | 1767.42 | 8.67 | 0.00 |

Table S16. AIC_c_ model selection for first-born pup mass at weaning (FB_mw_) as a function of pup sex, mother age, year of primiparity and offspring mass at weaning (MO_mw_, n= 113). The preferred model is in bold.

| Model | *K* | AIC_c_ | ∆AIC_c_ | *w_i_* |
| --- | --- | --- | --- | --- |
| **age+pupsex** | **12** | **815.79** | **0.00** | **0.52** |
| age | 11 | 817.34 | 1.55 | 0.24 |
| age+pupsex+ MO_mw_ | 13 | 818.07 | 2.28 | 0.17 |
| age+ MO_mw_ | 12 | 819.61 | 3.82 | 0.08 |
| age+pupsex+year | 21 | 832.62 | 16.83 | 0.00 |
| age+year | 20 | 833.67 | 17.88 | 0.00 |
| age+pupsex+ MO_mw_ + year | 22 | 835.71 | 19.92 | 0.00 |
| age+ MO_mw_ +year | 21 | 836.67 | 20.88 | 0.00 |
| intercept | 2 | 837.20 | 21.41 | 0.00 |
| Pupsex | 3 | 837.22 | 21.43 | 0.00 |
| MO_mw_ | 3 | 839.29 | 23.50 | 0.00 |
| MO_mw_ +pupsex | 4 | 839.35 | 23.56 | 0.00 |
| year+pupsex | 12 | 845.39 | 29.60 | 0.00 |
| year | 11 | 845.49 | 29.70 | 0.00 |
| MO_mw_ + year | 12 | 847.44 | 31.65 | 0.00 |
| pupsex+ MO_mw_ +year | 13 | 847.56 | 31.77 | 0.00 |

Table S17. Parameter estimates from GLM of birth date (days since Dec 1) as a function of maternal age (4-6, 7-8, 9+ yr) and recruited female’s length at weaning (MO_lw_).

| Coefficients | Estimate | SE | z-value | Pr(>\|z\|) |
| --- | --- | --- | --- | --- |
| (Intercept) | 6.94 | 17.05 | 0.41 | 0.6845 |
| age 7-8 | -4.6 | 1.51 | -3.02 | <0.01 |
| age 9+ | -14.3 | 3.52 | -4.08 | <0.001 |
| MO_lw_ | 0.2 | 0.15 | 1.59 | 0.116 |

Null deviance: 12069 on 162 degrees of freedom

Residual deviance: 10469 on 159 degrees of freedom

Table S18. AIC_c_ model selection for first-born birth date as a function of mother age, year, and offspring body length at weaning (MO_lw_, n= 163). Preferred models are in bold.

| Model | K | AICc | ∆AICc | *w_i_* |
| --- | --- | --- | --- | --- |
| **momage + MO_lw_** | **9** | **1157.86** | **0.00** | **0.56** |
| **momage** | **8** | **1158.43** | **0.57** | **0.42** |
| year | 11 | 1168.22 | 10.35 | 0.00 |
| year+ MO_lw_ | 12 | 1168.28 | 10.42 | 0.00 |
| intercept | 2 | 1168.31 | 10.45 | 0.00 |
| MO_lw_ | 3 | 1169.87 | 12.01 | 0.00 |
| year+momage | 17 | 1170.82 | 12.96 | 0.00 |
| year + momage + MO_lw_ | 18 | 1170.88 | 13.02 | 0.00 |

Table S19. AIC_c_ model selection for age groups to include in the model of first-born birth date (since Dec 1) as a function of maternal age at primiparity and offspring body length at weaning (MO_lw_; FP_bd_ ~ momage + MO_lw_). Preferred model is in bold

| Age Factors | *K* | AIC_c_ | ∆AIC_c_ | *w_i_* |
| --- | --- | --- | --- | --- |
| **4&5&6,7&8,9+** | **5** | **1151.42** | **0.00** | **0.60** |
| 4&5&6,7,8,9+ | 6 | 1153.57 | 2.15 | 0.21 |
| 4&5,6to8,9+ | 7 | 1155.72 | 4.30 | 0.07 |
| 4to9+ | 8 | 1155.85 | 4.42 | 0.07 |
| all | 9 | 1157.86 | 6.44 | 0.02 |
| 4&5&6&7,8,9+ | 5 | 1158.20 | 6.77 | 0.02 |
| 4to8+ | 7 | 1159.03 | 7.61 | 0.01 |

Table S20. AIC_c_ model selection for first-born birth date and mother age, year, and offspring length (MO_lw_) or mass at weaning (MO_mw_, n=78). Preferred model is in bold.

| Model | K | AICc | ∆AICc | *w*_i_ |
| --- | --- | --- | --- | --- |
| **momage** | **8** | **565.61** | **0.00** | **0.48** |
| momage**+** MO_lw_ | 9 | 567.17 | 1.56 | 0.22 |
| momage+ MO_mw_ | 9 | 567.86 | 2.26 | 0.15 |
| intercept | 2 | 569.76 | 4.15 | 0.06 |
| year | 11 | 570.96 | 5.36 | 0.03 |
| MO_mw_ | 3 | 571.87 | 6.27 | 0.02 |
| MO_lw_ | 3 | 571.91 | 6.30 | 0.02 |
| year+ MO_lw_ | 12 | 573.42 | 7.81 | 0.01 |
| year+ MO_mw_ | 12 | 573.71 | 8.10 | 0.01 |
| year+momage | 17 | 584.10 | 18.49 | 0.00 |
| year+momage+ MO_lw_ | 18 | 587.01 | 22.41 | 0.00 |
| year+momage+ MO_mw_ | 18 | 587.38 | 21.77 | 0.00 |
